# Phase distribution of chronic myeloid leukemia in Bangladesh

**DOI:** 10.1186/1756-0500-7-142

**Published:** 2014-03-13

**Authors:** Md Abdul Mottalib, Tanvira Afroze Sultana, Md Ibrahim Khalil, Siew Hua Gan, Md Sirazul Islam, Subhagata Choudhury, M Anwar Hossain

**Affiliations:** 1 Laboratory Services Division, Bangladesh Institute of Research and Rehabilitation for Diabetes Endocrine and Metabolic Disorders (BIRDEM), 122 Kazi Nazrul Islam Avenue, Shahbag, Dhaka, Bangladesh; 2 Department of Biochemistry and Molecular Biology, Jahangirnagar University, Savar, Dhaka, Bangladesh; 3 Human Genome Centre, School of Medical Sciences, Universiti Sains Malaysia, Kubang Kerian, Kelantan 16150, Malaysia; 4 Department of Biochemistry and Molecular Biology, University of Dhaka, Dhaka, Bangladesh

**Keywords:** Chronic myeloid leukemia, Bangladesh, RT-PCR, Philadelphia chromosome

## Abstract

**Background:**

Here, we report the phase distribution of chronic myeloid leukemia (CML), defined based on the World Health Organization criteria, among 63 patients in Bangladesh. All patients were diagnosed based on complete blood count, bone marrow examination including bone marrow aspiration and reverse-transcriptase polymerase chain reaction (RT-PCR). Out of 63 patients, 42 were male and 21 were female. The mean age of the subjects was 37.4 years, with an age range of 17-60 years. The majority of patients (86%) were classified in the chronic phase (CP), 7 (11%) in the accelerated phase (AP) and two (3%) in blast crisis (BC). The most frequent patient age ranges were 21-30 years for CP, 41-50 years for AP and 41-50 years for BC.

**Results:**

The Philadelphia chromosome was detected in 48 patients by RT-PCR. The mean total leukocyte counts, platelet counts, hemoglobin levels and marrow blast frequencies were 101 × 10^9^/L, 409 × 10^9^/L, 12.2 g/dl and 2.8% for CP; 121 × 10^9^/L, 418 × 10^9^/L, 8.7 g/dl and 15% for AP and 311 × 10^9^/L, 396 × 10^9^/L, 9.2 g/dl and 26% for BC, respectively.

**Conclusion:**

This study concluded that most CML patients in Bangladesh are from a younger age group (31-40 years). In addition, males were more commonly affected, although females were afflicted with this disease at a younger age.

## Background

Chronic myeloid leukemia (CML) is a clonal bone marrow stem cell disorder characterized by the proliferation of mature granulocytes (neutrophils, eosinophils and basophils) and their precursors. This myeloproliferative disease is associated with a characteristic chromosomal translocation called the Philadelphia chromosome, which is a balanced reciprocal translocation involving chromosomes 9 and 22. The generation of the Philadelphia chromosome is associated with the formation of the break point cluster region-Abelson (BCR-ABL1) chimeric gene, which is associated with increased tyrosine kinase activity and is currently thought to play a central role in the pathogenesis of CML [1].

Historically, CML diagnosis include only complete blood count, which comprises differential and platelet counts, marrow aspiration and marrow biopsy. However, recent developments in CML diagnosis have led to more sensitive tests, such as standard cytogenetics, fluorescent *in situ* hybridization (FISH) and quantitative polymerase chain reaction (PCR) [2]. It has been reported that cancer causes approximately 12% of all mortalities worldwide [3]. In the developed world, cancer is the second leading cause of death after cardiovascular disease and accounts for 21% (2.5 million) of all mortalities. In the developing world, cancer is ranked the third leading cause of death and accounts for 9.5% (3.8 million) of all mortalities [3].

The major risk factors for CML include occupational exposure to benzene and high doses of ionizing radiation [4], which was evidenced by the 20- to 25-fold increased incidence of leukemias observed among atomic bomb survivors [5]. However, limited data are available regarding other risk factors for CML, such as alcohol abuse [6], obesity and weight gain during adulthood [4], which requires additional study. There is no data available on the effect of preservatives or pesticides used in the food industry on CML.

Historically, CML was treated with chemotherapies such as hydroxyurea plus interferon (a family of naturally occurring proteins produced by cells of the immune system). However, the key breakthrough in the treatment of this disease began in the late 1980s and early 1990s with the introduction of bone marrow transplantation [7]. This was followed by the launch of a drug named imatinib in the late 1990’s as a first-line therapy, which dramatically improved the survival of patients. In fact, recent studies have reported the 10-year survival rate of CML to be approximately 80% [8,9]. Such marked improvements in the management of CML have resulted in decreased mortality rates for certain populations, mainly those in developed countries [10]. However, in developing countries and particularly in India, there have also been recent improvements in CML diagnosis, with genetic testing available in some centers since 2001. The availability of improved tests may result in reducing the rate of misclassifications for leukemias and will likely improve CML incidence and mortality rates.

However, few epidemiological reports have addressed whether such improvements in CML diagnosis have taken place in Bangladesh. Bangladesh is an overcrowded country with 160 million inhabitants and the population is steadily increasing. The majority of the population lives in villages (60 to 70%) and the majority of citizens are illiterate. Moreover, the availability of health care is non-uniform, with medical facilities at par with the best in the world in the metropolitan areas and large cities but leaving residents in rural areas largely deprived of basic medical support. As a result, only a very small minority of patients exposed to chronic diseases is covered by health care policies. To our knowledge, this is the first report to assess the distribution of the three phases of CML in Bangladesh.

Bangladesh is recently facing a serious threat of food adulteration. Various chemicals not approved by Bangladesh Standards and Testing Institution (BSTI), the regulatory body of Bangladesh, are being used all over the country to increase the shelf life of natural and few manufactured food items. Among the various illegal preservatives, the use of formalin which is the soluble form of formaldehyde is most widespread. Formalin is applied on fish for preservation; calcium carbide on fruits to ripen; brick dust in chilli powder; urea to whiten rice and puffed rice; sawdust in loose tea; soap in Ghee; and artificial sweetener, coal tar, and textile dyes in sweetmeats. More than 76 percent food items on the market were adulterated based on a random survey by Public Health Laboratory of Dhaka City Corporation in 2004. According to another survey conducted by the National Toxicology Program of the United States, statistically significant increased risks (RR = 1.42, 95% CI = 0.92 to 2.18) were found for all lymphohematopoietic cancers combined, for leukemias combined, and for myeloid leukemia among persons occupationally exposed to any form of formaldehyde including formalin. Relative risks increased with increasing peak exposure [11]. To date, there is no epidemiological survey of various cancers in Bangladesh or the impact of this chronic exposure to carcinogens throughout the population reported.

As CML is a relatively silent disease with a usually delayed presentation and a long survival period, we selected this group of hematological malignancies to study its trend in our country. To date, there is still no established cancer registry available in Bangladesh. Furthermore, data preservation in the hospitals is also at a very primitive level. The study was therefore limited to whatever documents available of patients or examined at our molecular diagnostic laboratory for the presence of BCR-ABL1. This study may provide an insight into the pattern of haematological malignancies in Bangladesh in the face of such chronic exposure to formalin and other carcinogenic materials in food.

## Methods

The study was conducted in the Hematology Department of the Bangladesh Institute of Research and Rehabilitation for Diabetes Endocrine and Metabolic Disorders (BIRDEM), Shahbag, Dhaka between June 2006 and May 2010. Samples were collected from outpatient clinics and inpatients suspected to be suffering from blood cancer. In this study, all newly diagnosed CML patients (based on hematological profile) older than 17 years of age were included.

### Inclusion criteria

•Age ≥ 18 years

•Signed and dated informed consent form prior to protocol-specific screening procedures

•Cytogenetic- or PCR-based diagnosis of any phase of Ph + CML

•Adequate duration of prior therapy

•ECOG Performance Status of 0 or 1 for chronic phase patients

•No antiproliferative or antileukemia treatment within 7 days of the first dose of imatinib or hydroxyurea

•Able to take daily oral capsules or tablets reliably

### Exclusion criteria

•Ph chromosome - negative or BCR-ABL - negative CML

•Pregnant or breastfeeding

•Prior history of allergies to imatinib

A detailed history was obtained for each patient, and questionnaires and physical examinations were also administered Additional file 1. Investigations included complete blood counts with differential and bone marrow aspirations, which were conducted by a consultant hematologist. This study protocol was approved by the institutional ethics committee of Bangladesh Institute of Research and Rehabilitation for Diabetes Endocrine and Metabolic Disorders (BIRDEM), Dhaka, Bangladesh and conducted in accordance with the Helsinki Declaration. Informed consent was obtained from all the subjects.

PCR was performed by a senior scientific officer in the Haematology Unit of the General Laboratory at BIRDEM. PCR was conducted using a nested reverse-transcriptase polymerase chain reaction (RT-PCR) method. Specific primers were incorporated to identify common fusion transcripts of chromosomes 9 and 22 in the region of q34;q11 (Table 1). To detect the presence of the BCR-ABL1 gene or Philadelphia chromosome, mRNA was extracted from 63 of the suspected CML patients. The mRNA was converted to cDNA by a reverse transcriptase. After confirming the presence of cDNA using a housekeeping gene (B actin), the cDNA was further amplified by PCR.

**Table 1:** PCR primer sequences

**Gene**		**Name**	**Primer sequence**	**Product size (bp)**	**Positive control cDNA**
BCR-ABL1	1^st^	BCR1	5′-GCTTCTCCCTGACATCCGTG-3′	1840	KT1 1840 (e13a2) B3 2415 (e14a2)
		ABL1	5′-GGCCCATGGTACCAGGAGTG-3′	2415	
	2^nd^	BCR2	5′-GGAGCTGCAGATGCTGACCAAC-3′	446	
		ABL2	5′-GTTTCTCCAGACTGTTGACTG-3′	371	

The total cellular RNA was isolated from bone marrow or peripheral blood samples containing between 2 × 10^6^ and 1 × 10^7^ leucocytes, as determined using a commercial kit (PureLink™ Purification Kit, Version B, Invitrogen, USA) according to the manufacturer’s recommendations. The concentration of RNA was estimated spectrophotometrically and the ratio of the optical density at 260 and 280 nm was calculated. Only samples with a ratio between 1.80 and 2.00, indicating good quality RNA, were included.

*In vitro* reverse transcription of 1 μg or less of total RNA to complementary DNA (cDNA) was performed in a 20 μl volume containing Quantiscript reverse transcriptase and a mixture of both oligo-dT and random hexamers as primers; a commercial kit (QuantiTect Reverse Transcription Kit; QIAGEN GmbH, Germany) was used according to the manufacturer’s recommendations. The reverse transcriptase included in the kit consists of a mixture of the Omniscript and Sensiscript reverse transcriptases, and this transcriptase was added to the PCR mixture containing 1.5 mmol/L magnesium chloride, 50 mmol/L potassium chloride, 10 mmol/L Tris-hydrochloride (pH 8.3), 200 μmol/L dNTP, 2.5 U of HotStarTaq Polymerase and 400 nmol/L of primers. The PCR conditions for each individual primer set are indicated in Table 2. A nested PCR method was followed. Amplification was initially carried out for 30 cycles using an outer set of primers, then 1 μl of the PCR product was used to perform a second round of 30 cycle amplification using a different set of primers designed inwards to the first pair.

**Table 2:** PCR conditions for BCR-ABL1

**Hot-start PCR**	**15 min**	**95°C**
3-step cycling		
Denaturation	30 s	94°C
Annealing	30 s	55°C
Extension	1 min	72°C
Number of cycles	30	
Final extension	1 min	72°C

A positive and a negative control were used with every batch of PCR reaction. For positive control, cDNA sample of a previous case that was found to be positive for BCR-ABL1 in our lab, was used at the same concentration as the test case. For negative control, either sample from a patient with no evidence of BCR-ABL1 gene or DNA free-PCR grade water was used. The test case was reported as positive only when the negative control did not show any bands while the positive control showed a band at the same position, thus ensuring the absence of contamination as well as the effectiveness of the PCR run.

Finally, 10 μl of PCR product was run on a 2% agarose gel (molecular biology grade) in 1 × TAE buffer at 100 V for a maximum of 90 min, which was stained with ethidium bromide prior to visualization under a UV transilluminator (Optima UVP, Japan) at 365 nm. Either the cell lines or cDNA from samples previously found positive for the respective translocations were used as positive controls. To ensure the presence of good-quality cDNA, the amplification of β-actin mRNA was consistently performed with 1 μl of a similar batch of cDNA preparation used to identify the respective translocations.

After completion of the investigation, patients were categorized into various CML phases based on the World Health Organization (WHO) criteria [12]. The chronic phase (CP) was defined as myeloid blasts less than 10% in the peripheral blood or bone marrow. The accelerated phase (AP) was defined as blasts 10-19% of white blood cells in peripheral and/or nucleated bone marrow cells; persistent thrombocytopenia (< 100 × 109/L) unrelated to therapy or persistent thrombocytosis (> 1000 × 109/L) unresponsive to therapy; increasing white blood cells and spleen size unresponsive to therapy and or cytogenetic evidence of clonal evolution. Blast crisis (BC) phase was defined as peripheral blood blasts ≥ 20% of peripheral blood white blood cells or nucleated bone marrow cells; extramedullary blast proliferation; and large foci or clusters of blasts on bone marrow biopsy.

### Statistical analysis

The data were analyzed using SPSS for windows version 10 (Statistical Packages for Social Science, IBM Corporation, New York, USA). The numerical data, such as age and laboratory variables including hemoglobin (Hb), total leucocyte count, platelet count and blast frequency, were reported as the mean ± S.D. Categorical data such as gender, genotype and CML phase were expressed as frequencies (percentage).

## Results and discussion

During the 2-year study period, 48 patients between the ages of 17 and 68 years were successfully included (Table 3). The majority (67%) were males, with 33% females and a male to female ratio of 2:1. The majority of patients were in the 31- to 40-year-old age bracket. The mean age was 38.73 years for males and 36.60 years for females, with an overall mean age for both sexes of 37.4 years.

**Table 3:** Age and sex distribution of the CML patients

**Age group years**	**Male**	**Percentage (%)**	**Female**	**Percentage (%)**	**Total**	**Percentage (%)**
15-20	2	6.25	1	6.25	3	6.25
21-30	10	31.25	3	18.75	13	27.08
31-40	7	21.88	8	50	15	31.25
41-50	4	12.5	2	12.5	6	12.5
51-60	8	25	2	12.5	10	20.8
> 60	1	3.13	0	0.00	1	2.08
Total	32		16		48	

### Hematological data

The hematology data for the three CML phases are listed in Table 4.

**Table 4:** Hematological data

	**Value**	**CP (N = 39)**	**AP (N = 7)**	**BC (N = 2)**
1	Hb (g/dl)			
	Range	6.0-14.0	5.6-11.8	7.2-11.2
	Mean ± SD	11.81 ± 2.90	8.75 ± 2.13	9.20 ± 2.80
	Number of patients with a Hb level:			
	< 10 g/dl	16	5	1
	10-12 g/dl	24	2	1
	> 10 g/dl	14		
2	TLC (×10^9^/L)			
	Range	12-452	21-300	180-443
	Mean ± SD	101.70 ± 99.70	121.28 ± 93.66	311.50 ± 185.90
	Number of patients with TLC			
	< 50	19	2	0
	50-150	25	3	0
	> 150	10	2	2
3	Platelets (×10^9^/L)			
	Range	135-1,000	700-800	393-400
	Mean ± SD	409.65 ± 228.50	418.42 ± 261.00	396.50 ± 4.90
	Number of patients with a platelet count			
	< 150	4	2	0
	> 150	50	5	2
4	Blast cell (%)			
	Range	0-9	11-19	23-30
	Mean ± SD	2.73 ± 2.23	15.00 ± 3.26	26.50 ± 4.94
	Number of patients with blast cell %			
	< 9	54	0	0
	10-19	0	7	0
	> 20	0	0	2

TLC = total leukocyte count.

### CML phase distribution

48 patient samples were PCR-positive for the BCR-ABL1 fusion gene (Table 5). The majority of the patients (81.25%) were in the CP stage, with the majority of these patients between 31.40 years of age (Table 6). For AP, the majority of patients were between 41-50 years of age, while the majority of BC patients were between 31-50 years of age.

**Table 5:** Results for RT-PCR

**RT-PCR findings**	**Number of patients**	**Percentage (%)**
PCR-positive	48	76.19
PCR-negative	15	23.81
Total	63	100.00

**Table 6:** CML phase distribution

**Age**	**CP**	**Percentage (%)**	**AP**	**Percentage (%)**	**BC**	**Percentage (%)**
15-20	2	5.13	1	14.28	0	0.00
21-30	12	36.77	1	14.28	0	0.00
31-40	13	33.33	1	14.28	1	50.00
41-50	2	5.13	3	42.85	1	50.00
51-60	9	23.08	1	14.28	0	0.00
>60	1	2.56	0	0.00	0	0.00
Total	39	81.25	7	14.58	2	4.17

This is the first study to report the distribution of CML phases in Bangladesh. In general, males (67%) were affected more frequently than females (33%); the male to female ratio was 2.1:1.0, which strongly correlates with previous data from India (male: female ratio of 1.9:1.0) [13]. In addition, this ratio is slightly higher than that reported in Pakistan (male: female ratio of 1.7:1.0) [14] or male: female ratio of 2.0:1.0 [15] but slightly lower than that reported in the West Indies (male:female ratio of 2.4:1.0) [16]. The high male to female ratio in our study may be attributed to the lower socio-demographic status in Bangladesh. Furthermore, the care providers to female patients are less likely to consider this disease on their differential if a female patient presents with signs and symptoms of CML. However, it is also possible that the biological mechanism of this disease is also a potential cause and cannot be disregarded. The diagnostic situation in Bangladesh is further compounded by the high cost of PCR analysis, and other socio-cultural factors may lead to the reluctance of female patients to pursue tests such as rt-PCR; this may be a source of bias leading to an underrepresentation of the true disease burden in women.

When the study population was grouped based on age, the highest frequency of CML cases was observed in the 31- to 40-year-old age group (range 17-68 years). The mean age of male CML patients was 38.73 years, but the mean age for females was lower (36.60 years). The mean age of both sexes was 37.68 years, which is lower than the mean ages previously reported in Pakistan (38 years) [17], India (43 years) [13], France (55 years) [18] and the USA (66 years) [19]. In our opinion, there is no single identifiable factor that can explain the elevated incidence of CML among younger individuals in Bangladesh. Our study provides the basis for future research seeking to explore the risk factors responsible for this early onset of CML. However, potential contributing factors for the earlier occurrence of CML in our population may be due to the consumption of food contaminated with dye (painting grade), fish contaminated with formalin, bananas contaminated with carbide and chemically preserved mangos, which are common local delicacies. Furthermore, the overuse of pesticides and insecticides to increase crop yield may also be a contributing factor that should be investigated further. The above mention factors are lower or absent in Europe and North America. Moreover, the percentages of younger populations afflicted with CML are higher in Bangladesh, Pakistan and India when compared to Europe or in North America. Therefore, it may be postulated that there is a delay in the onset of CML in Europe or North America or there could be genetic predisposition in the Asian countries which warrants further investigations.

The frequency of all three phases of CML at the time of clinical presentation was 81.25%, 14.58% and 4.17% for CP, AP and BC, respectively. To the best of our knowledge, this study is the first to report the phase distribution of CML in Bangladesh. In comparison, the study by Riaz Ahmed *et al.*[17] from Rawalpindi, Pakistan reported the frequencies of the three phases of CML to be 77.8%, 15.5% and 6.7% for CP, AP and BC, respectively. Another study conducted in Pakistan, which included 275 cases of CML, revealed the frequencies of CP, AP and BC cases to be 87.3%, 8.1% and 4.7%, respectively. In addition, a large multi-center study in France reported the frequencies of CP, AP and BC as 96.8%, 2.2% and 0.9%, respectively, at the time of diagnosis [18]. Therefore, our data strongly correlate with the data previously reported for Pakistan [20] and one possible explanation for this similarity may be that Pakistan is a neighboring country with similar ethnic demography and sociocultural factors.

Nevertheless, there were some limitations to our study. Although BIRDEM is a tertiary care hospital, it specializes mainly in diabetic care with less emphasis on cancer treatment. Additionally, in this study, patients were sampled from only a single hospital, which may not represent the actual clinical scenario in Bangladesh. We also did not analyze patients based on ethnic group, socioeconomic background or educational status due to the scarce data available on leukemia and poor record keeping. However, we hope that the findings from this study will encourage and support future research addressing these issues.

This study is a very first step in understanding the patterns and distribution of CML in Bangladesh. Further investigations are necessary to understand the epidemiology, potential risk factors, biology and genetics of hematological malignancies in this country in rapid transition.

## Conclusion

This is the first report of the phase distribution of CML in Bangladesh. Out of 48 patients studied, the majority of patients (81.25%) were classified in the chronic phase (CP), seven (14.58%) in the accelerated phase (AP) and two (4.17%) in blast crisis (BC). The most frequent patient age ranges were 31.40 years for CP, 41-50 years for AP and 31-50 years for BC. The Philadelphia chromosome was detected in 48 patients by RT-PCR. This study concluded that most CML patients in Bangladesh are relatively young (31-40 years). In addition, males were more commonly affected, although females were afflicted with this disease at a younger age.

## Competing interests

The authors declare no competing interests.

## Authors’ contributions

This work was carried out in collaboration among all authors. MAM and TAS performed the experiments. MSI, SC and AH supervised the work and evaluated the results. MIK and GSH searched literatures and revised the manuscript. All authors read and approved the final manuscript.

## Supplementary Material

Additional file 1 The questionnaires of the CML study. Click here for file
