# Supplementary material for: Phase distribution of chronic myeloid leukemia in Bangladesh
Source: BMC Res Notes. 2014 Mar 13;7:142. doi: 10.1186/1756-0500-7-142 (PMC4008259; doi:10.1186/1756-0500-7-142)
Supplement: Additional file 1 — The questionnaires of the CML study. [file 1756-0500-7-142-S1.doc]

# Additional file 1: The questionnaires of the CML study

Provisional diagnosis …………………

Serial no. Present no…………………… Previous no………………………

Registration no (ID NO.)…………….. Date of Registration………………...

Name of the Patient…………………………………………………………………………

Age. ……Years.. ……. Months, Sex i) M ii) F

Referred By:…………………………………………………………………………….

Present Address : Village/ Road no./ House…………………………………………….

Post Office:

Thana:

District:

Mobile No………………………..

Permanent Address’ Village/ Road no./ House…………………………………………….

Post Office:

Thana:

District

**Date of Diagnosis**………./……/……..

**Clinical Presentation:**

i) At diagnosis ii) Follow-up

Complaints:

Fever:

Bleeding manifestations

Lymph node

Liver

Spleen

Others

**Present Investigation**:

| Date |  |  |  |  |  |  |  |
| --- | --- | --- | --- | --- | --- | --- | --- |
| Hb: |  |  |  |  |  |  |  |
| TC: |  |  |  |  |  |  |  |
| DC: |  |  |  |  |  |  |  |
| PC: |  |  |  |  |  |  |  |
| S Creat: |  |  |  |  |  |  |  |
| SGPT: |  |  |  |  |  |  |  |
| LDH: |  |  |  |  |  |  |  |

Bone Marrow: ……………………………………………………………………

Karyotyping:………………………………………………………………………

FISH:………………………………………………………………………………

Previous rtPCR: (date)…………………………………………………………..

**Treatment History**:

Conventional Chemotherapy:

Specific (Imatinib/ Enliven for BCRABL and ATRA/ Vasonoid for PML RAR&):

PCR Results

**Appendix-II**

**M/15 PHOSPHATE BUFFER SOLUTION:**

(Chairprost, 1996; Kent & Kubica, 1985)

**Stock Solutions:**

1. M/15 disodium phosphate

Dissolve 9.47 gm of anhydrous Na_2_HPO_4_ in distilled water to make one litter (1000ml)

2.M/15 monopotassium phosphate

Dissolve 9.07 gm of KH_2_PO_4_ in distilled water to make one litter (1000ml).

**PHOSPHATE BUFFER pH- 7.0**

Mix 61.1 ml of # (1) stock solution with 38.9 ml of # (2) Stock solutions.

Finally confirm pH with pH meter.
